# Cell Membrane Biomimetic Nano-Delivery Systems for Cancer Therapy

**DOI:** 10.3390/pharmaceutics15122770

**Published:** 2023-12-13

**Authors:** Zhenxing Xia, Weiwei Mu, Shijun Yuan, Shunli Fu, Yongjun Liu, Na Zhang

**Affiliations:** 1 NMPA Key Laboratory for Technology Research and Evaluation of Drug Products, Jinan 250012, China; 202136894@mail.sdu.edu.cn (Z.X.); weiweimu2137@mail.sdu.edu.cn (W.M.); 202016769@mail.sdu.edu.cn (S.Y.); 17854160137@163.com (S.F.); 2 Key Laboratory of Chemical Biology (Ministry of Education), Department of Pharmaceutics, School of Pharmaceutical Sciences, Cheeloo College of Medicine, Shandong University, 44 Wenhua Xi Road, Jinan 250012, China

**Keywords:** cancer therapy, cell membrane, biomimetic, nano-delivery systems

## Abstract

Nano-delivery systems have demonstrated great promise in the therapy of cancer. However, the therapeutic efficacy of conventional nanomedicines is hindered by the clearance of the blood circulation system and the physiological barriers surrounding the tumor. Inspired by the unique capabilities of cells within the body, such as immune evasion, prolonged circulation, and tumor-targeting, there has been a growing interest in developing cell membrane biomimetic nanomedicine delivery systems. Cell membrane modification on nanoparticle surfaces can prolong circulation time, activate tumor-targeting, and ultimately improve the efficacy of cancer treatment. It shows excellent development potential. This review will focus on the advancements in various cell membrane nano-drug delivery systems for cancer therapy and the obstacles encountered during clinical implementation. It is hoped that such discussions will inspire the development of cell membrane biomimetic nanomedical systems.

## 1. Introduction

Cancer is a deadly disease that seriously threatens the health of millions of people. According to statistical data, there will be an estimated 19.3 million newly diagnosed cases of cancer on a global scale in 2020, resulting in around 10 million cancer-related fatalities [1,2]. Traditional cancer treatment methods, such as surgery, chemotherapy, and radiotherapy, have led to increased survival rates among cancer patients [3,4,5]. However, traditional therapies hardly target tumor cells to exert therapeutic effects, and off-target drugs may induce injury to the body’s normal tissues. As a result, the treatment process can produce adverse or even fatal side effects [4,6,7,8,9,10]. Nanomedicine delivery systems such as liposomes, micelles, inorganic nanoparticles, and polymeric nanoparticles have been developed and utilized in the treatment of cancer [7,11,12]. These systems enable passive or active targeting of tumor sites to enhance the efficacy of cancer therapy [13,14,15,16,17,18].

However, in our study, passively targeting tumors merely by utilizing nano-delivery system particle size, surface charge properties, high permeability of the tumor site, and enhanced permeability and retention (EPR) effects were found to be difficult to obtain the desired accumulation of tumor tissues [15,19,20,21]. Phagocytosis by the endothelial system, construction of protein crowns, and blood clearance hinder the delivery of the nanomedicine system to the tumor [22]. Active targeting of tumor tissues by ligand-modified nano-delivery systems has been investigated, but single ligand-modified nano-delivery systems make it difficult to achieve ideal active targeting in the treatment of certain tumor diseases [18,23,24]. To improve the delivery efficiency of nanodrug systems, researchers have developed some enhancement strategies. For example, modification of polyethylene glycol (PEG) on the surface of the nano-delivery systems creates a site barrier to mask the surface charge and prolong the circulation time of the formulation [25,26,27,28,29,30]. Modification of multiple ligands on the surface of nano-delivery systems improves the ability to target tumors actively [16]. However, studies have shown that repetitive PEG modifications may accelerate blood clearance and fail to achieve long circulation in vivo [31]. The development of multiligand-modified nano-delivery system technology is also limited by the complexity and poor reproducibility of the preparation process [32,33]. In addition, artificially modified nanoparticles are considered foreign substances in the body and can be easily recognized and cleared by the body, making it difficult to achieve the desired therapeutic effect [34,35]. Therefore, the construction of a nano-delivery system with good biocompatibility, long blood circulation time, and strong tumor-targeting ability is the focus of research.

Cell membrane biomimetic nanomedicine delivery systems present a new approach to address these issues. Cells serve as the fundamental building blocks of the body and play vital roles in supporting regular life functions. Each cell possesses distinct and remarkable abilities. Red blood cells (RBC) serve as transporters in the circulatory system and can be circulated in the body for extended periods. Platelets can adhere to the matrix and play a crucial role in the body’s coagulation process in various diseases. The cancer cells have homologous targeting abilities and the antigens can stimulate the immune response in the body [36,37,38]. The cell membrane retains these functions through a variety of biomacromolecules present on the membrane. In 2011, the first cell membrane biomimetic nanomedicine system was developed by Zhang et al. They coated polymer cores with red blood cell membranes, allowing the nanoparticles to take advantage of the prolonged circulation of red blood cells and their ability to avoid clearance by macrophages in the blood. As a result, the nanoparticles remained in circulation for approximately 40 h and achieved remarkable therapeutic effects [39]. The success of biomimetic nano-delivery systems for erythrocyte membranes has led to the development of biomimetic nano-delivery systems. A variety of cell membranes has been developed for the preparation of biomimetic nano-delivery systems, including platelet, leukocyte, cancer cell, and stem cell membranes, etc. Significant improvements have been made in the biocompatibility and tumor-targeting of the nano-delivery systems. Further research on biomimetic nano-delivery systems for cell membranes, and genetically engineered and hybrid membranes have been developed to enhance the efficacy of cancer therapy [35,40]. 

Therefore, this paper focuses on the research progress and preparation techniques of cell membrane biomimetic nanomedicine drug delivery systems (CMBNDS) based on different types of cell membranes. Their important role in cancer therapy and the existing research and clinical challenges are discussed. A contribution to the development of cell membrane bionic nano-delivery systems is expected.

## 2. Preparation and Characterization of Cell Membrane Biomimetic Nanomedicine Delivery Systems

The CMBNDS is composed of a nanoparticle core and an externally modified source cell membrane, prepared through three main steps: (1) acquiring the source cell membrane, (2) synthesizing the nanoparticle core, and (3) preparing the CMBNDS [41]. The synthesis of nanoparticle cores can be found in the literature, so this review focuses on methods for obtaining source cells and preparing the CMBNDS. Then, the methods for characterizing the successful preparation of the CMBNDS are summarized. 

### 2.1. Source Cell Membrane Acquisition

Cell membranes are obtained in two steps: cell membrane extraction and cell membrane purification [42]. During this process, it is critical to maintain membrane integrity and purity. The higher the membrane integrity and purity, the better the function of the source cells obtained by CMBNDS [43,44]. Therefore, choosing the right extraction and purification method is critical to ensure in vivo safety [45]. Detailed summaries of the most commonly used methods for extracting cell membranes and their advantages and disadvantages are given in Table 1.

Hypotonic lysis is the creation of a low osmotic pressure environment outside the cell. Cells swell and rupture as they absorb water. It is widely used in erythrocyte membrane extraction because of its simplicity [55,56]. Zhang et al. obtained healthy red blood cells from fresh blood samples of mice and then utilized the hypotonic method to acquire red blood cell membranes. Consequently, they modified red blood cell membranes on the surface of nanoparticles. The successful preparation was characterized by the results of transmission electron microscopy (TEM) [39]. However, hypotonic methods exhibit lower efficiency in cell membrane recovery and are rarely used for other cell membrane extractions. Repeated freezing and thawing are widely used in platelet membrane extraction due to their straightforwardness, but the functionality of cell membrane surface proteins can be affected as a result of cyclical exposure to cold and heat [50]. Ultrasound destroys cell membranes through powerful external forces. The heat generated by this process may affect the activity of proteins. Therefore, the system temperature needs to be stabilized during operation [57]. Chemical lysis is mainly applied to large quantities of material within cells and is rarely used to extract cell membranes [51,52]. Homogenization can obtain compliant cell membranes with adjustable pressure levels. It applies to large-scale cell membrane extraction and has great potential for industrial applications [53,54]. Each cell membrane extraction method has its advantages and disadvantages. Therefore, the extraction method should be selected according to different needs when preparing CMBNDS.

The main methods for the purification of cell membranes are differential centrifugation and ultrafiltration. Centrifugation can be used to obtain a cell membrane precipitate for Akaryote [58]. The sedimentation coefficients of complex organelle and nuclear membranes vary in nucleated cells. Cell membranes are generally isolated by differential centrifugation [59]. The main application is for samples with large differences in density. Ultrafiltration is a more accessible and less density-specific approach compared to differential centrifugation. However, it is constrained by the sample size and is difficult to apply to large-scale production [60]. Therefore, when selecting extraction and purification methods for cell membranes, it is crucial to consider the nature of various cells and the production scale. Employing suitable techniques is essential to achieve superior quality and purity of cell membranes.

### 2.2. CMBNDS Preparation

Cell membrane-encapsulated nanoparticles are a crucial aspect of CMBNDS formation. The methods used to integrate cells into the nanoparticle core mainly include physical co-extrusion, sonication, and more advanced microfluidic electroporation techniques.

#### 2.2.1. Co-Extrusion

**Figure 1:**
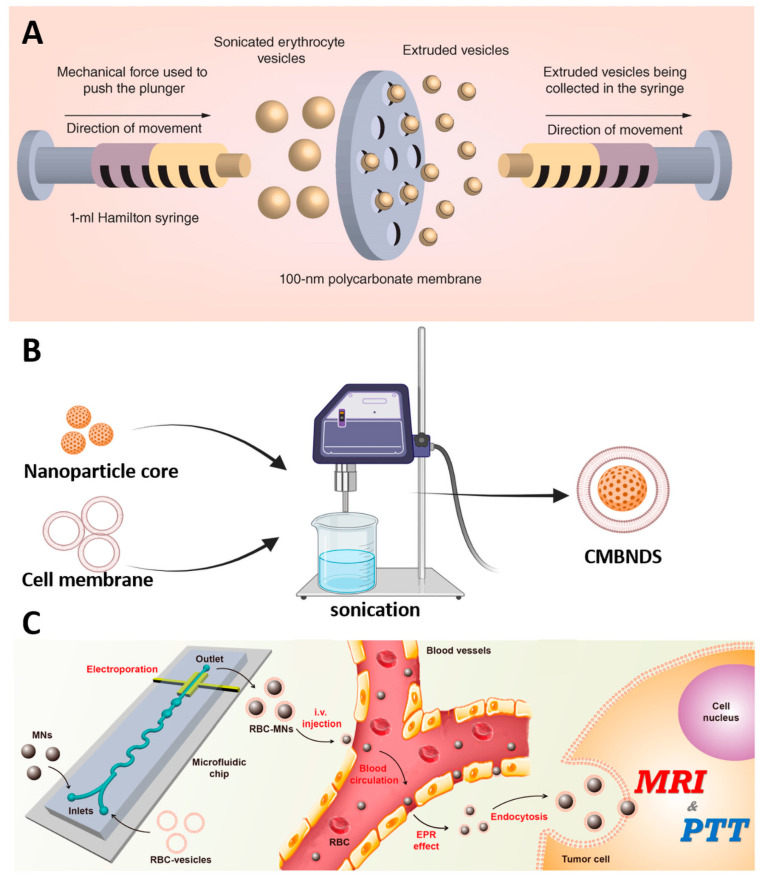
Preparation method of CMBNDS. (**A**) Co-extrusion method. Reprinted with permission from Ref. [63] 2016, MDPI. (**B**) Sonication method. Created with Biorender.com. (**C**) Microfluidic electroporation method. Reprinted with permission from Ref. [64] 2017. American Chemical Society.

Co-extrusion is the physical extrusion of a mixture of nanoparticles and cell membranes through the nanopores of a polycarbonate membrane [61]. The fluidity of the cell membrane enables it to reorganize around the nanokernel while extruding, resulting in the formation of a cell membrane-nanoparticle structure (Figure 1A). Liu and colleagues prepared R-RBC@ DACHPt@ indocyanine green (ICG)@anionic bovine serum albumin nanoclusters (aBSA NCs) (BPtI) through the repeated extrusion of a mixture containing 1,2-diamino cyclohexane-platinum (DACHPt) and red blood cell membranes. The structure of R-RBC@BPtI was observed using transmission electron microscopy, revealing a regular spherical core and a 10 nm cell membrane surrounding the exterior. The particle size was 167 nm and the surface potential was −12.8 mV, which is similar to the cell surface potential [62]. The size of cell membrane coated (CMC)-NPs is reliant on the diameter of the membrane pores. The co-extrusion approach generates superior homogeneity and controlled particle size distribution in comparison to the alternatives. However, this technique is burdensome, time-intensive, and expensive.

#### 2.2.2. Sonication

Unlike co-extrusion, sonication produces dispersed cell membranes by acoustic energy. When cell membranes were co-incubated with nanoparticle cores, CMBNDS of uniform particle size were produced (Figure 1B). The power, frequency, and duration of ultrasound were adjusted to optimize the preparation efficiency [65]. Copp et al. achieved RBC membrane encapsulation by fusing RBC membrane vesicles with PLGA particles for 36 min using sonication at a frequency of 42 kHz and power of 100 W. The transmission electron microscopy (TEM) results indicated that the preparation possessed a well-defined core–shell structure [66]. Ultrasonication offers the benefit of being user-friendly and appropriate for laboratory synthesis. However, this process generates heat. It is important to ensure the preservation of the structural integrity and functionality of the biofilm during the preparation process.

#### 2.2.3. Microfluidic Electroporation

The microfluidic electroporation and ultrasound methods share similar principles [67]. In a microfluidic device, Rao et al. injected Fe_3_O_4_ magnetic nanoparticles (MN) and RBC membrane-derived vesicles (RBC-vesicles) into microfluidic devices (Figure 1C) [64]. As the compound passes through the electroporation zone, the RBC vesicles are punctured by the electrical pulse to create small holes, allowing the MN to enter and form a structure of red blood cell membranes-encapsulated MN (RBC-MN). Finally, RBC-MN was collected from the outlet and demonstrated to possess long blood circulation properties in vivo. Microfluidic electroporation was shown to effectively promote the synthesis of CM-NPs. This method offers the advantage of delicate control over the membrane coating process through adjustable parameters. It is possible to obtain CMBNDS with complete coatings and improved quality.

### 2.3. Characterization of CMBNDS

After the successful synthesis of CMBNDS, the physicochemical properties and biological functions of CMBNDS need to be evaluated, and then the ex vivo and in vivo drug delivery capabilities need to be further examined to confirm that the cell membrane is correctly encapsulated on the surface of the nanoparticle core and that it can inherit the unique capabilities of the cell. 

#### 2.3.1. Physicochemical Properties

**Figure 2:**
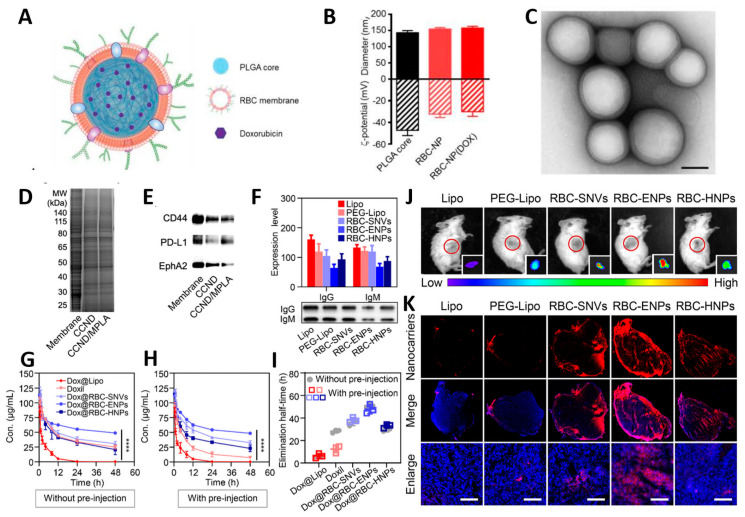
Characterization of CMBNDS. (**A**) The structure of RBC-NP. (**B**) The size and surface potential of RBC-NP. (**C**) The TME images of RBC-NP. Reprinted with permission from Ref. [68] 2016, MDPI. (**D**) SDS-PAGE results. (**E**) Western blotting of CD44, PD-L1 and EphA247. Reprinted with permission from Ref. [70] 2023, MDPI. (**F**) Assay of IgG and IgM adsorbed in the protein corona of RBC-derived nanocarriers and liposomes in vivo. (**G**,**H**) Blood circulation curves. (**I**) Related elimination half-life. (**J**) Representative images of 4T1-bearing mice and ex vivo images of tumors. (**K**) Representative CLSM images. Scale bar: 100 μm. Reprinted with permission from Ref. [71] 2017. **** *p* < 0.0001, American Chemical Society.

The changes in particle size and surface potential of CMBNDS during preparation can characterize the success of the preparation, and the physical appearance of CMBNDS can be observed by TME. The core–shell structure of the CM-NP results in a noticeable change in particle size when compared to non-encapsulated nanoparticle cores (Figure 2A). Similarly, the surface potential of the CM-NP will display results similar to those of the cell membrane’s surface potential (Figure 2B) [68]. The size and surface potential of particles provide preliminary characterization for synthesizing CM-NP. TME presents a more intuitive approach to determining CM-NP synthesis. TME images can observe the core–shell structure of CM-NP (Figure 2C). Therefore, it can be used as a visual indicator that the cell membrane completely encapsulates the inner core of the nanoparticles [69].

#### 2.3.2. Biological Function

Preliminary evidence for the successful encapsulation of nanoparticles in cell membranes was obtained by studying the physicochemical properties of CMBNDS. Furthermore, it is necessary to determine that the cell membrane coating effectively preserves biomolecules and functional structures essential for cellular function. The primary techniques employed for biological function characterization include sodium dodecyl sulfate-polyacrylamide gel electrophoresis (SDS-PAGE) and Western blotting, which are also standard approaches for assessing membrane protein characterization on CMBNDS. Guo et al. performed SDS-PAGE and Western blotting after encapsulating nanodiscs in cancer cell membranes and observed whether they possessed a protein profile similar to that of MC38 plasma membranes (Figure 2D) [70]. Western blotting analysis confirmed the presence of the tumor-promoting marker CD44, programmed death ligand 1 (PD-L1), and the tumor-associated antigen EphA247 (Figure 2E). These results indicated that the tumor cell membrane-coated nano-discs successfully inherited the homologous targeting and immune stimulation abilities of tumor cells. Further, the optimization effect of modified cell membranes on the function of the nano-delivery system was investigated. In vivo and in vitro, the abilities of CMBDNS to overcome blood circulation clearance and enhance tumor drug delivery were examined. To investigate the effect of RBC membrane modification on liposome delivery function, Miao and his colleagues intravenously injected mice with PEG-modified liposomes (PEG-Lipo) and RBC membrane-modified liposomes (RBC-lipo). Compared with PEG-Lipo, RBC-lipo adsorbed fewer proteins to the surface, had the lowest IgG/IgM levels, and could effectively avoid blood clearance (Figure 2F) [71]. The in vivo blood circulation time study showed that the RBC membrane provided the liposomes with a longer circulating half-life (51.1 h), enabling long-term circulation of the nano-delivery system in the blood (Figure 2G–I). Injection of RBC membrane-modified liposomes in vivo to verify the ability of RBC HNPs to accumulate at the tumor site by fluorescence in vivo imaging in mice showed good accumulation of RBC HNPs at the tumor site (Figure 2J). CLSM results show that the fluorescence of RBC-HNPs spread throughout the tumor, demonstrating stronger tumor penetration compared to PEG-Lipo (Figure 2K). Other cell membranes besides RBC membranes can similarly enhance the tumor drug delivery capability of nano-delivery systems, and validation of the relevant capabilities of CMBNDS can confirm that the cell membranes are correctly encapsulated on the surface of the nanoparticle cores and inherit the unique capabilities of the cells.

## 3. Classification of CMBNDS

**Figure 3:**
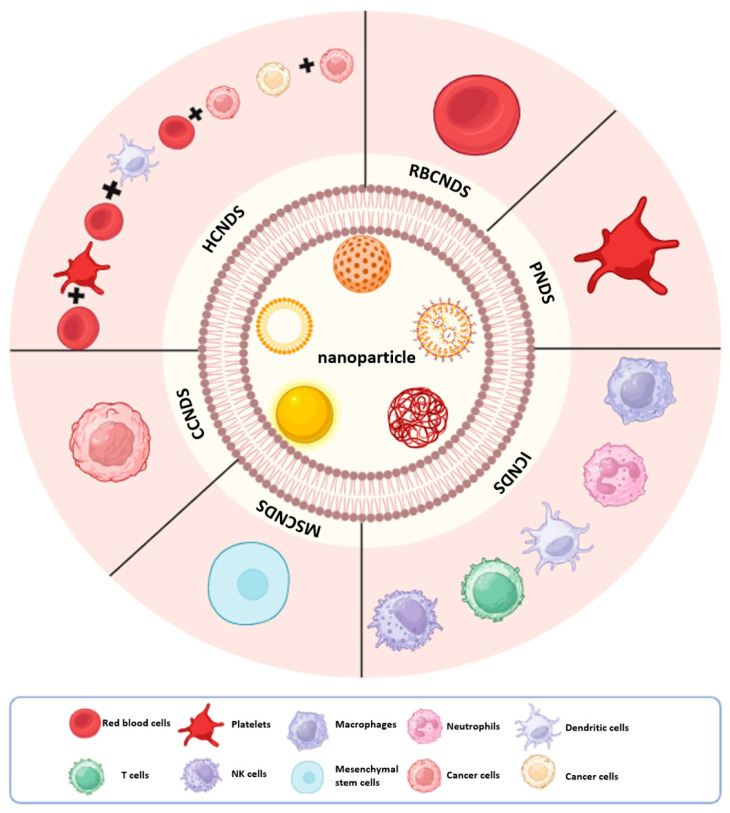
Different types of CMBNDS. Cell membranes can be collected from different kinds of cells and then used to coat the various nanoparticles, yielding CMBNDS. Created with Biorender.com.

The primary challenge of in vivo drug delivery for cancer therapy is maintaining drug stability in the bloodstream. CMBNDS has achieved remarkable results in addressing this issue. For example, after surface modification of red blood cell membranes with nanoparticles (NPs), the delivery system can evade recognition and clearance by the immune system and has a longer circulation time in the body [72]. Additionally, other cell membranes in the body have unique capabilities that can help in cancer therapy, including immune evasion, tumor-targeting, penetration of the blood–brain barrier, and uptake by tumor cells [46,72,73]. Therefore, an increasing number of CMBNDS utilizing various cell membranes have emerged in recent years and displayed remarkable efficacy in cancer therapy. This paper will present the latest research advancements in CMBNDS over recent years and evaluate their potential for application in cancer therapy across six types of modified cell membranes: (1) Red blood cell membrane biomimetic nano-delivery system; (2) Platelet membrane biomimetic nano-delivery system; (3) Immune cell (Macrophages, Neutrophils, Dendritic cells, T cells, Nature Kill (NK) cells) membrane biomimetic nano-delivery system; (4) MSC membrane biomimetic nano-delivery system; (5) Cancer cell membrane biomimetic nano-delivery system; and (6) Hybridized cell (RBC-Platelet, RBC-Cancer cell, Immune cell-cancer cell, Cancer cell-Cancer cell) membrane biomimetic nano-delivery system (Figure 3).

### 3.1. Red Blood Cell Membrane Biomimetic Nano-Delivery System (RBCNDS)

Red blood cells are abundant in the blood, with research indicating that their number approaches 30 trillion in the human body. Their lack of nuclei and organelles is advantageous in extracting red blood cell membranes to prepare CMBNDS. A crucial factor in the immune system’s ability to detect and remove foreign bodies is the presence of “self-labeling” in host cells of the human body. Red blood cell membranes naturally express a variety of immunomodulatory markers, including CD47, a transmembrane protein that binds to inhibitory receptor signaling regulator alpha. CD47 inhibits the phagocytosis of red blood cells by immune cells and promotes the circulation of red blood cells in the human body for up to 120 days [74]. Thus, using red blood cell membranes to create a biomimetic nano-delivery system can result in prolonged in vivo circulation and drug clearance avoidance [75].

**Figure 4:**
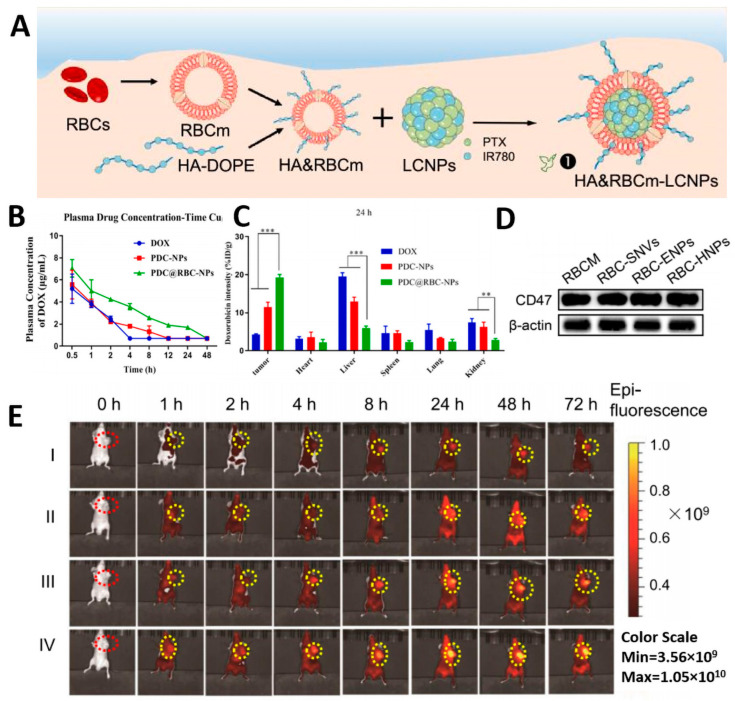
(**A**) The preparation of HA&RBCm-LCNPs, Reprinted with permission from Ref. [77] 2021, MDPI; (**B**) Mean plasma concentration-time profiles of free DOX (2.5 mg/kg), PDCNPs, and PDC@RBC-NPs (2.5 mg/kg of DOX); (**C**) Tissue distribution and tumor accumulation of PDC@RBC-NPs in BEL-7402 cell tumor-bearing mice. ** *p* < 0.01, *** *p* < 0.001. Reprinted with permission from Ref. [76] 2022, Elsevier; (**D**) Retained the CD47 protein. Reprinted with permission from Ref. [71] 2022, MDPI; (**E**) In vivo, biodistribution of HA&RBCm-LCNPs Reprinted with permission from Ref. [77] 2021, BMC.

Modifying NPs with PEG can increase colloidal stability and extend circulation time in vivo, improving the therapeutic effects against cancer. However, repeated administration of PEG-modified NPs accelerates blood clearance, making it difficult to achieve desired outcomes. Inspired by the prolonged circulation of RBCs in the blood, Wang et al. enhanced in vivo hepatocellular carcinoma (HCC) efficacy by coating NPS with RBCm to extend blood circulation [76]. The red blood cell membranes-encapsulated pectin-adriamycin mimetic nanoparticles developed through hypoosmotic chromatography and mechanical co-extrusion. The results indicate that pectin-doxorubicin (PDC)@RBC-NPs were more stable in serum than Doxorubicin (DOX) and PDC-NPs (Figure 4B). Administration of PDC@RBC-NPs with prolonged circulating properties significantly increased accumulation at tumor sites in mice with hepatocellular carcinoma by nearly three-fold compared to free DOX (Figure 4C), suggesting improved therapeutic efficacy. Similarly, to achieve extended blood circulation capability, Miao and colleagues developed nanoparticles of polyethylene glycol diacrylate (PEGDA) hydrogel encapsulated in red blood cell membranes using ultrasonic techniques. Drug release during in vitro blood circulation simulations was less than 30% within 8 h. Moreover, the cell membrane coating efficiently retained the CD47 protein (Figure 4D), indicating the acquisition of red blood cell-like long-acting circulation capability by these nanocarriers [71].

However, RBCNDS suffer from low drug-loading capacity and lack of tumor-targeting ability. The inner core of CMBNDS is the primary site of drug packaging. The drug loading capacity of RBCNPs can be effectively improved by increasing the encapsulation capacity of the core. Zhang et al. constructed HA&RBCM-coated lipid multi-compartment nanoparticle cores (HA&RBCm-LCNPs) (Figure 4A). Its unique honeycomb internal structure can improve drug loading capacity. The encapsulation efficiency of paclitaxel (PTX) and IR780 was 95% and 93%, respectively [77]. Furthermore, RBCNPs can utilize the EPR effect to passively accumulate at the tumor site, but this targeting ability is uncertain. Improving their ability to target tumors is crucial in enhancing the therapeutic impact against cancer. To address this, Pan et al. developed an engineered red blood cell membrane (RBCm) coated with red rhodopsin/indocyanine green nanovesicles (ARISP) [78]. Modifying RBC coats with anti-low-density lipoprotein receptor (LDLR), which can specifically target tumor hypoxia sites through LDLR receptors overexpressed at hypoxic tumor sites. However, this strategy may have an impact on the proteins on the membrane and destroy the structure of the membrane, which needs to be carefully considered when adopting. On the other hand, utilizing the natural fluidity of the cell membrane can enable RBCNPs to target tumors while preserving cell membrane stability. Zhang and colleagues hybridized the natural CD44 receptor, hyaluronic acid (HA), to the surface of red blood cell membranes (RBCm) to enhance the tumor-targeting efficacy of the vector. The ability of hybridized RBCm to target tumors was increased fourfold compared to unhybridized RBCm (Figure 4E).

### 3.2. Platelet Membrane Biomimetic Nano-Delivery System (PNDS)

Platelets are small fragments of cytoplasmic debris produced by mature megakaryocytes in the bone marrow. Similar to red blood cells, platelets lack a nucleus and consist only of a cytoplasm surrounded by a plasma membrane. Typically, there are between 150,000 to 350,000 platelets per microliter in the blood. It is advantageous to extract cell membranes for the preparation of CMBNDS. In addition, they are stabilized in the human blood circulation for 7–11 days and express fewer proteins on the surface of the cell membrane, making them safer. As an indispensable component of the blood, platelets can accumulate in damaged or inflamed vascular endothelial sites through surface membrane biomolecules such as GPIbα, GPIa/IIa, GPVI, etc. Studies have shown that during cancer progression, vascular leakage or inflammation can stimulate platelet heterotypic adhesion to form a tumor thrombus. Simultaneous interaction of membrane proteins α6β1, αIIbβ3 and p-selectin with tumor cells promotes immune escape and tumor metastasis [79,80,81,82]. Therefore, the characteristics of platelet membranes suggest novel opportunities for cancer therapy [83].

**Figure 5:**
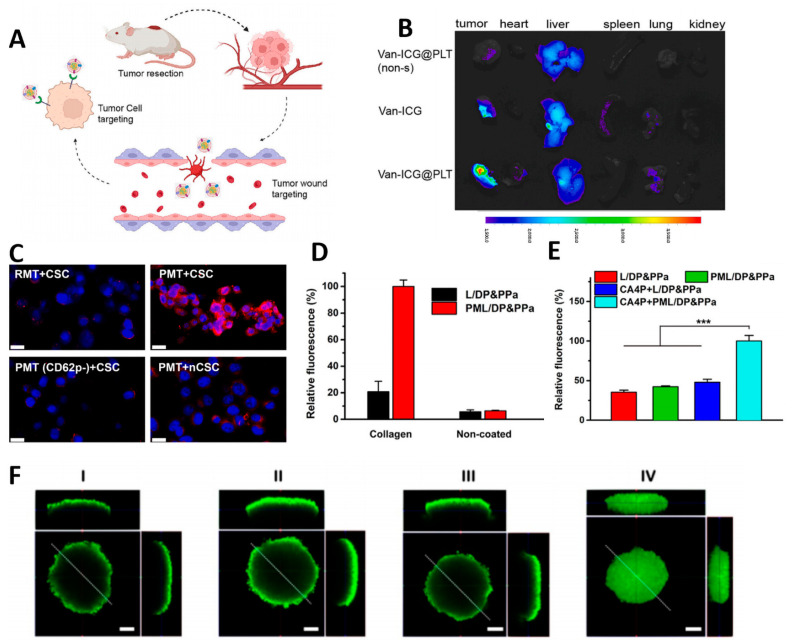
(**A**) The mechanism of cascade tumor-targeting. (**B**) Ex vivo fluorescence images of tumors and organs Reprinted with permission from Ref. [84] 2023, Elsevier; (**C**) CLSM images different nanoparticles. Scale bars: 20 μm. Reprinted with permission from Ref. [85] 2023; (**D**) Fluorescence quantification of D@SN-P at tumor neovascularization. (**E**) Fluorescence quantification of D@SN-P at the tumor site. Reprinted with permission from Ref. [86] 2022, MDPI; (**F**) The penetration ability of pH-HCM@FeCNDs in an acidic environment. Scale bar: 100 µm. Reprinted with permission from Ref. [87] 2022, Ivyspring International. *** *p* < 0.001.

For cancer patients who are eligible for surgical treatment, removing residual tumor cells at the surgical site post-operation offers significant benefits. Based on the property of platelets to aggregate at damaged blood vessels (Figure 5A), Liu et al. fabricated a biomimetic nanosensor system Van-ICG@PLT using platelet membranes [84]. The system has proven to be effective. Utilizing platelets’ innate orientation, vancomycin (Van) and indocyanine green (ICG) can be delivered to surgical incisions. Addressing the issue of off-target effects in TNBC treatment includes achieving accurate localization, preventing photosensitizer accumulation in normal tissues, reducing side effects and toxicity, and effectively eliminating residual tumor cells and bacterial infections. The in vivo study revealed that Van-ICG@PLT accumulated significantly better in postoperative tumors than in non-surgical tumors (Figure 5B). Within the postoperative tumor microenvironment, the p-selectin protein present on the surface of platelet membranes interacts positively with CD44, the corresponding surface receptor on 4T1 cells. This interaction promotes the recognition of tumor cells by Van-ICG@PL. The latter significantly decreased tumor growth in hormonal mice, resulting in a tumor suppression rate of approximately 83%.

P-selectin on the platelet membrane can specifically bind to CD44 on the surface of cancer stem cells (CSCs) to precisely target cancer cells and achieve tumor-targeting. In a study by Ning et al., platelet membrane-modified nanoparticles were used to precisely kill CSCs and prevent tumor recurrence after radiotherapy [85]. Platelet membrane proteins increase PNDS circulation time in vivo, promote immune escape, and improve therapeutic efficacy. The experimental results showed that PMT could successfully target CSC (Figure 5C). Additionally, the rate of tumor recurrence was reduced. Thus, PNDS is a therapeutic approach with desirable biosafety and significant potential for clinical translation. To enhance PNDS’s capacity for tumor-targeted accumulation, Ding et al. incorporated the tumor vascular disrupting agent 5,6-dimethylxanthone 4-acetic acid (DMXAA) into the supramolecular nanomedicine (SN) and coated its surface with a platelet membrane to produce a platelet-mimicking supramolecular nanomedicine (D@SN-P) [88]. After entering the body, D@SN-P initially accumulates in tumors by leveraging the platelet membrane’s ability to accumulate at tumor neovascularization (Figure 5D). The release of DMXAA disrupts tumor vasculature, further doubling the accumulation of D@SN-P at the tumor site (Figure 5E).

PNDS also suffers from difficulty in drug release. To tackle this issue, Yang et al. utilized platelet membranes (CM) and pH-responsive vesicles (pH-Vs) to create pH-responsive hybrid cell membranes (pH-HCM), which were then wrapped around FeCNDs to produce pH-responsive biomimetic nanoclusters. pH-HCM@FeCNDs [87]. pH-HCM@FeCNDs effectively target tumor cells and release therapy agents in response to pH for deeper penetration and combined thermo-chemical therapy. In vivo experiments confirm its ability to penetrate the acidic environment of orthotropic breast cancer, leading to tumor reduction (90.33%) and minimal metastatic effects (0.29%) (Figure 5F). 

### 3.3. Immune Cell Membrane Biomimetic Nano-Delivery System (ICNDS)

Immune cells are commonly present in blood vessels, lymphatic vessels, and other tissues, with diameters ranging from 7 to 20 μm. The different types include neutrophils, dendritic cells, macrophages, eosinophils, T cells, and NK cells. They identify inflammation and accumulate in diseased regions. Chronic inflammation is a prominent feature of cancer, where massive inflammatory cells can be recruited by tumor-produced cytokines and chemokines to promote tumor progression [89,90,91]. The inflammatory chemotaxis inherent in immune cell membranes has emerged as a prominent strategy for targeted drug delivery to tumors. In the following section, we will highlight the advancements made in five nano-delivery systems that mimic different leukocytes, specifically macrophages, neutrophils, dendritic cells, T cells, and NK cells.

#### 3.3.1. Macrophage Membrane Biomimetic Nano-Delivery System (MNDS)

During tumor progression, the macrophages are recruited to the tumor site as a type of inflammatory cell [20,21]. Upon arrival at the tumor, macrophages receive tumor-secreted cytokines to transform from M1-type macrophages with tumor-suppressing capabilities into M2 macrophages that promote tumor growth [20,92]. Tumor tropism in macrophages presents novel avenues for targeted delivery of antitumor drugs using nanotechnology [21,93]. Specifically, the biomolecules on the surface of macrophage membranes enable them to evade phagocytosis by immune cells and can bind specifically to tumor site receptors. Therefore, nanoparticles enclosed in the macrophage membrane can evade quick clearance by the reticuloendothelial system (RES) and the mononuclear phagocyte system (MPS), and penetrate deeply into tumors [94].

**Figure 6:**
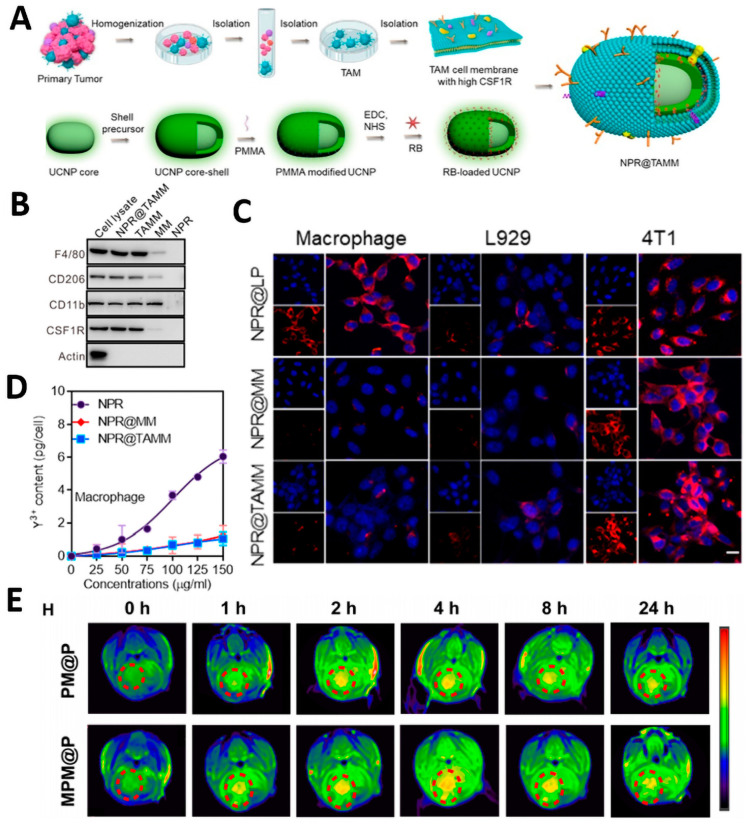
(**A**) Schematic illustration of the preparation of TAMM-coated NPR@TAMMs. (**B**) Representative image of SDS gel showing protein bands of different nanoparticles and whole cells. (**C**) In vitro function of NPR@TAMMs. Scale bar: 50 μm. (**D**) Avoid macrophage phagocytosis. Reprinted with permission from Ref. [95] 2023, MDPI. (**E**) Schematic illustration of the in vitro BBB model. Reprinted with permission from Ref. [97] 2023, American Chemical Society.

The tumor-homing and immune-compatibility characteristics of tumor-associated macrophage membrane (TAMN) inspired Chen et al. to develop NPR@TAMM, which consists of TAMN-encapsulated upconverting nanoparticles (Figure 6A) [95]. The TAMN coating retains specific protein markers that are overexpressed on TAM (Figure 6B). NPR@TAMMs have been found to possess a stronger ability to target tumor cells when compared to NPR@LPs (Figure 6C). After the addition of NPR@TAMMs, macrophages exhibited reduced phagocytic ability (Figure 6D), suggesting that the TAMN coating effectively prevents nanoparticle phagocytosis. In vivo therapeutic results demonstrated that NPR@TAMMs were able to eliminate primary tumor growth and produce a therapeutic effect of inhibiting distant tumor growth. Similarly prepared macrophage-camouflaged nanoparticles also have good tumor-targeting ability [96]. 

The tumor propensity of the macrophage membrane and receptor-mediated endocytosis of endothelin-2 peptide could help the nano-delivery system to efficiently cross the Blood Tumor Barrier (BTB) and target Glioblastoma Multiforme (GBM) regions. Therefore, Xiao et al. formed nano gels (NGs) by precipitation polymerization, which surface-modified macrophage membranes loaded with MnO_2_ and cisplatin. For in situ chemotherapy/chemodynamic therapy (CDT) of glioma [97]. The modified nanoparticles were able to effectively promote the nanoparticles to cross the BTB and induce apoptosis in cancer cells through the interaction of integrin α and β1 on the surface of the cell membrane (Figure 6E). Biomimetic nano-delivery platform modified by macrophage membrane can not only prolong the blood circulation time to avoid blood clearance but also specifically target tumors and enhance the deep delivery of drugs, providing a new idea for tumor treatment.

#### 3.3.2. Neutrophil Membrane Biomimetic Nano-Delivery System (NNDS)

Neutrophils are the most prevalent innate immune effector cells with the ability to target the inflammatory environment at the tumor site through inflammation-oriented mechanisms [98]. Chu et al. developed a tumor-targeting nano-delivery platform with a polymeric nitroimidazole core encapsulated within neutrophil membranes [99]. Nanoparticles camouflaged with neutrophil membrane are recruited to the pre-metastatic niche (PMN) during tumor metastasis. Additionally, neutrophils possess cell-intrinsic adhesion molecules that enable them to selectively target circulating tumor cells. The final neutrophil membrane-encapsulated nanoparticles accumulated 2.4 times stronger than bare nanoparticles at the PMN. Exploiting Interactions Between Neutrophils and Circulating Tumor Cells. Wu et al. developed immunomagnetic nanoparticles (IMNs) functionalized with surface-encapsulated neutrophil membranes. Reduced nanoparticle interaction with immune cells, enhances stability in the circulation, and interaction with circulating tumor cells (CTCs) improves the ability to isolate them. [100]. The results showed that the separation efficiency of neutrophil membrane-encapsulated IMNs (Neu-IMNs) ranged from 41.36% to 96.82%, and the purity ranged from 40.25% to 90.68% compared with that of bare IMNs. The ability to efficiently isolate CTCs is a great inspiration for early diagnosis of cancer and precision medicine.

#### 3.3.3. Dendritic Cell Membrane Biomimetic Nano-Delivery System (DCNDS) 

Dendritic cells (DCs) are responsible for antigen presentation in vivo and play an important role in initiating, regulating, and maintaining the immune response [101,102]. They recognize and internalize tumor antigens, process them intracellularly, and expose them on the cell surface as antigenic peptide major histocompatibility complex (pMHC) molecules that are presented to T lymphocytes and activate antigen-specific immunity to eliminate tumor cells [103,104]. For this reason, Xu and colleagues created a hybrid nano-vaccine (Hy-M-Exo) by merging tumor-derived exosomes (TEX) and dendritic cell membrane vesicles (DCMV) to develop a potent immune response against cancer [105]. The resulting hybrid nano-vaccine membrane retains CCR7, a key protein for DCMV lymphatic homing, and can effectively target the lymph node (LN). It also maintains tumor antigens and endogenous signals on the membrane, which can trigger an active T-cell response, significantly activating the immune system and impeding tumor growth. DCNDS demonstrate high feasibility and significant clinical value in activating anti-tumor immunotherapy. Additionally, DC membrane proteins can facilitate BTB traversal for cells, while antigen-presenting complexes can activate CD8+ T cells and induce remodeling of the tumor immune microenvironment. Ma et al. created a nanoplatform, aDCM@PLGA/RAPA, which is encapsulated by an activated mature dendritic cell membrane (aDCM) that can cross the BTB and enhance the immune microenvironment [106]. In vitro, cellular uptake and trans-well assays demonstrated that aDCM@PLGA/RAPA effectively penetrated the BTB to target tumor cells through antigenic homology. Immunological analysis revealed that it leverages the antigen-presenting capability of the cell membrane to spur immature DCs toward maturation. This culminates in the activation of tumor-infiltrating T cells, NK cells, and other immune cells which subsequently initiate an immune response. Thus, the biomimetic nano-delivery system utilizing DC cell membrane has the potential to stimulate the body’s autoimmunity, resulting in a noteworthy inhibition of glioma growth.

#### 3.3.4. T Cell Membrane Biomimetic Nano-Delivery System (TCNDS)

Compared to expensive and complicated in vitro processing required for adoptive T cell therapy, TCNDS offers a simpler alternative by utilizing T cell membrane encapsulated nanoparticles to inherit some of the T cell functionalities [107,108,109]. These functionalities include active targeting of tumor tissues and activation of immune responses [110]. Since the cells are already deceased, TCMNPs do not experience immunosuppressive effects in the tumor microenvironment. However, they can promote the activation of immunosuppressive cells in the tumor microenvironment, resulting in outstanding tumor therapeutic effects [111]. Encapsulation of T cell membranes overexpressing PD-1 on DSF/Cu2+-loaded MXene nanosheets forms the CuX-P system [112]. The CuX-P system demonstrates a superior targeting effect in the presence of T cell membranes compared to nanosheets alone in both in vitro and in vivo tumor experiments. Notably, the T-cell membrane coating enriched with PD-1 continuously depletes PD-L1 on the tumor cell surface, making this system well-suited for tumor therapy and targeting. Like other cell membrane mimetic nano-delivery systems, TCNDS can be modified to enhance the ability to target tumor cells. Wu et al. developed a nano-delivery system that effectively targets glioblastoma cells. They genetically engineered T cells in vitro to produce a chimeric antigen receptor CAR structure targeting CD133 and EGFR. The modified cell membranes were then encapsulated on the surface of the nanoparticles to create CM@AIE NPs [113]. CM@AIE nanoparticles actively target tumor cells and penetrate the blood–brain barrier akin to immune cells, reaching the deeper glioma layers where they induce photothermal therapy effects.

#### 3.3.5. Natural Killer Cell Membrane Biomimetic Nano-Delivery System (NKCNDS)

Natural killer (NK) cells can recognize and eliminate tumor cells [114]. Like other immune cells, the membranes of NK cells can be used to develop nano-delivery systems that mimic the cell membranes [115]. These natural NK cell-like systems can target tumor cells specifically and avoid phagocytosis. A study showed the modification of NK cell membranes on mesoporous silica surfaces [116]. NK cell membranes provide good immune evasion for nano-delivery systems. After incubation with macrophages for 4h, the phagocytosed nanoparticles were barely visible in the cells. The proteins present in the cell membrane were characterized and the results showed that AsHMS-TA/FeIII@NK retained the original functional proteins and structure of NK. The in vivo findings revealed a two-fold improvement in the tumor-targeting ability of nanoparticles encapsulated by NK cell membranes. Integrins on the membrane surface of NK cells can interact with adhesion molecules on endothelial cells to disrupt the BTB opening a pathway to gliomas. Zhang and colleagues have developed a modified treatment to improve the therapeutic efficacy of glioma treatment. This entails using glioma-targeting ligand cRGD modification based on NK cell membranes, encapsulated on the surface of nanoparticles [117]. This system crosses the blood–brain barrier first in the presence of NK cell membranes after reaching the BTB, and then targeting tumor cells via specific ligands. Activate the immune response in the tumor tissue at the tumor site to exert the tumor treatment effect.

### 3.4. Mesenchymal Stem Cell Membrane Biomimetic Nano-Delivery System (MSCNDS)

Mesenchymal stem cells (MSCs) are multipotent stromal cells that can differentiate into various types of tissue cells under appropriate stimulation. These cells can be extracted from various tissues and retain their biological stem cell properties even after successive passages and cryopreservation [118,119]. MSCs have been widely studied due to their remarkable immunocompatibility, easy in vitro amplification, and tumor affinity, making them a highly sought-after biomimetic nano-delivery vector [120]. Studies have shown that MSCs can bind to tumor cells. This process is similar to the mechanism by which immune cells are recruited by tumors. The overexpressed cytokines and chemokines bind to the receptors on the cell membrane to recruit MSCs to the tumor site [121,122,123,124]. This distinctive characteristic suggests that MSCs are a potential and efficacious tool for targeting tumors in drug delivery.

**Figure 7:**
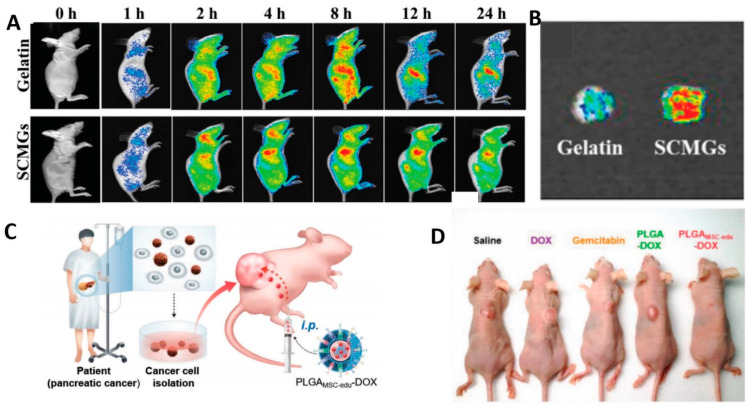
In vivo tumor-targeting ability of SCMGs. (**A**,**B**) Ex vivo images of the tumor. Reprinted with permission from Ref. [125] 2016, MDPI. (**C**) Schematic images for administration of educated MSCs membrane-coated PLGA–DOX. (**D**) Photographs of tumor-bearing mice after treatment. Reprinted with permission from Ref. [126] 2023, Wiley Blackwell.

The MSCS membrane is effective for targeting tumor cells for drug delivery. Modification of gelatin nanogels (SCMGs) with bone marrow-derived mesenchymal stem cell membranes. SCMGs exhibit excellent cancer-targeting ability in vivo after tail vein injection of cy7 -SCMG in mice compared to uncoated nanogels (Figure 7A,B), resulting in increased accumulation at the tumor site [125]. Similarly, the incorporation of mesenchymal stem cell membranes onto the exterior of polylactic acid-hydroxyacetic acid (PLGA) nanoparticles was shown to considerably enhance the accumulation of nanoparticles at the tumor location and refine the ability of DOX to exterminate the tumor [124]. Preprocessing of MSCs enhances their ability to target cancer cells. After adding stem cells to plates of cancer cells for culture, the expression of proteins targeting tumors on the stem cell membranes increased. Significantly enhanced ability to target cancer cell surface proteins. These treated stem cells are called educational stem cells. Intravenous injection of stem cell membrane-coated biodegradable polymer (polylactic acid-co-glycol, PLGA) nanoparticles (Figure 7C). The results showed that the nanoparticles significantly enhanced the targeting ability of pancreatic tumor cells and eliminated deep pancreatic tumor tissues (Figure 7D) [126].

### 3.5. Cancer Cell Membrane Biomimetic Nano-Delivery System (CCNDS)

Cancer cells have inherent immune evasion and tumor-targeting capabilities and can be readily acquired through in vitro cell culture, qualifying them as prospective constituents for the development of biomimetic nano-delivery systems to enhance tumor therapy [59]. The immune escape ability of cancer cells is mediated by overexpression of CD47 on the cell membrane. Moreover, cancer-homing properties are associated with cancer cell membrane surface adhesion molecules (CCAM), such as selectins, calreticulin, integrins, immunoglobulin superfamily (Ig-SF), and lymphocyte homing receptors [127,128,129,130,131]. These cancer cell capabilities can be inherited by CCNDS. This provides a new direction for the development of Biomimetic nano-delivery systems [132]. 

**Figure 8:**
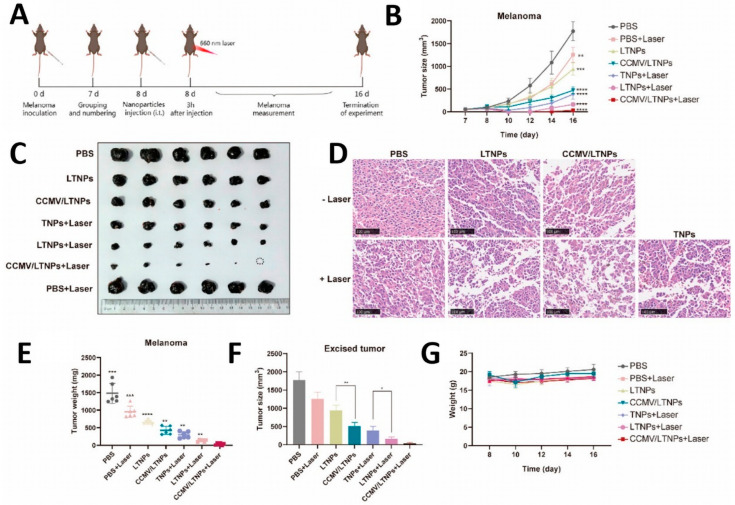
Antitumor efficacy. (**A**) Schematic of drug administration. (**B**) Tumor volume. (**C**) Photographs of tumors. (**D**) H&E staining images of the tumor (scale bar = 100 μm). (**E**,**F**) Stripped tumor weights and volumes. * *p* < 0.05, ** *p* < 0.01, *** *p* < 0.001, **** *p* < 0.0001. (**G**) Body weight of mice. Reprinted with permission from Ref. [134] 2023, American Chemical Society.

Cui et al. used cancer cell membranes to surface camouflage a multifunctional molecule with aggregation-induced emission properties (DHTDP) [133]. Benefits from abundant functional proteins and retention of membrane structure, it has significant homologous targeting ability, a longer half-life in blood circulation, and better cellular uptake. Photodynamic therapy (PDT) can instantly kill tumor cells by generating reactive oxygen species (ROS). The ability to target tumors ensures that PDT can have a chance to kill tumor cells stopping residual tumor cells from metastasizing. On the same principle, tumor cell membranes were encapsulated with nanoparticles coloaded with PDT, TLR7 agonists, and tumor antigens (CCMV/LTNPs) [134]. The cell membrane coating did not affect drug loading and remained stable in fetal bovine serum for a long time. By homologous targeting to the tumor cell membrane, CCMV/LTNP specifically targets and is endocytosed by tumor cells, showing outstanding anti-tumor ability (Figure 8).

Moreover, the CCNDS has excellent applications in tumor vaccines. As tumors express the CD47 molecule’s “do not eat me” signal on their surface, they evade clearance by the immune system. Blocking this pathway promotes the uptake of APCs into tumor cells and facilitates the presentation of tumor antigens. However, the application of anti-CD47 molecules to tumor therapy causes systemic side effects. To explore a more proven therapeutic approach, Liu et al. integrated CD47KO/CRT dual bioengineered B16F10 cancer cell membranes and unmethylated cytosine-phosphate-guanine (CpG) adjuvant to construct a vaccine [135]. By co-delivering antigens and immune adjuvants through tumor cell membranes, DBE@CCNPs promote mouse bone marrow-derived dendritic cell (BMDC) maturation and antigen cross-presentation. Combined anti-PD-L1 antibody avoids immune checkpoint molecule-induced T cell dysfunction and enhances tumor immunotherapy. Although CCNDS have shown promising results in tumor therapy, their translatability for clinical application requires further investigation of carcinogenic risks.

### 3.6. Hybridized Cell Membrane Biomimetic Nano-Delivery System (HCNDS)

With the development of CMNDS, the functions assigned to nanoparticles by a single-cell membrane cannot meet the needs of tumor therapy. Inspired by the enhanced delivery capacity of specific molecules modified on the cell membrane surface. Utilizing the fluidity of cell membranes to fuse two different cell membranes confers richer functionality to the nanoparticles. HCNDS can inherit specific capabilities from cells of both origins [136,137]. Thus, many HCNDS are used to enhance cancer therapy. This subsection will summarize the different hybrid membrane combinations and their ability to empower biomimetic nano-delivery systems to overcome the barriers of tumor therapy.

#### 3.6.1. RBC-Platelet Membrane Biomimetic Nano-Delivery System

**Figure 9:**
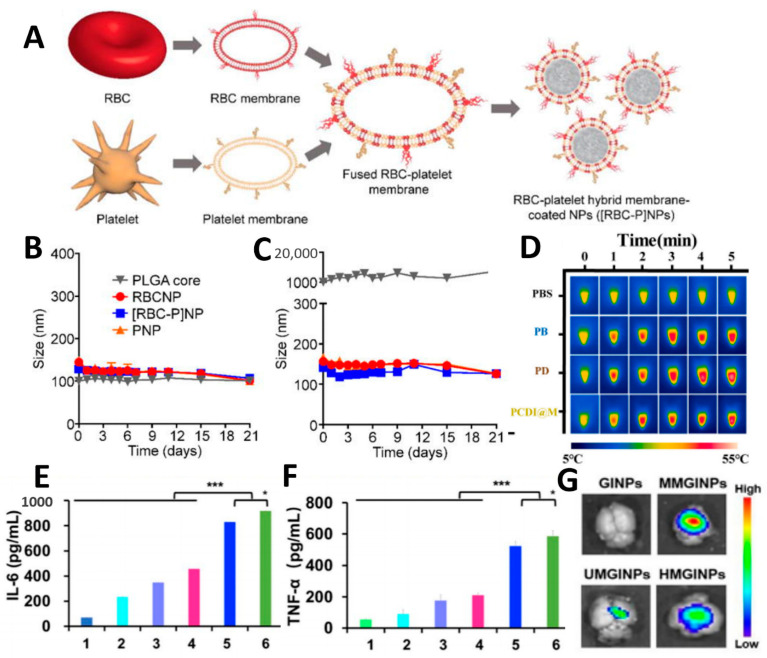
(**A**) Fabrication of RBC-platelet hybrid membrane-coated nanoparticles. (**B**,**C**) Nanoparticle stability in plasma. Reprinted with permission from Ref. [138] 2017, MDPI. (**D**) Photothermal curves of PCDI@M with different concentrations. Reprinted with permission from Ref. [139] 2021, ELSEVIER. (**E**,**F**) Immune stimulation efficiency. * *p* < 0.05, *** *p* < 0.001. Reprinted with permission from Ref. [140] 2021, MDPI. (**G**) The BBB penetrability. Reprinted with permission from Ref. [141] 2023, WILEY-V C H VERLAG GMBH.

The functional proteins and structural units of red blood cell membranes and platelet membranes are different, but both exhibit prolonged blood circulation. Therefore, co-assembling the two cell membranes may achieve a longer blood circulation time (Figure 9A). The results were promising, as the blood circulation time of RBC-platelet hybrid membranes was prolonged by 9.4 h and 13.5 h compared with that of RBC and platelet membranes alone, respectively (Figure 9B,C) [138]. Consequently, the hybrid membrane system offers the potential for better oncologic outcomes. In addition, the inflammatory and broken-vessel targeting properties possessed by platelets provide tumor-targeting possibilities for this hybrid membrane system. Although this has not been studied yet, it is worth investigating further.

#### 3.6.2. RBC-Cancer Cell Membrane Biomimetic Nano-Delivery System figure 

In addition to dual membrane-encapsulated NPs prepared by fusion of RBC and platelet membranes, other different cell membrane combinations such as RBC membranes and tumor cell membranes have received widespread attention for their excellent tumor therapeutic benefits. After preparing Hela cell membranes by membrane protein extraction, a mixture containing red blood cell membranes and Hela cell membranes (1:1 ratio) was sonicated for 10 min and incubated for 4 h to obtain red blood cell membranes–Hela cell membrane hybrid membranes by Xiao et al. RBC–Hela hybrid membrane was successfully coated on PCDI (PCDI@M) [139]. It can prolong the blood circulation time of nanocomposites and improve tumor-targeting, effectively killing tumor cells by photothermal therapy (Figure 9D). 

#### 3.6.3. Immune Cell-Cancer Cell Biomimetic Nano-Delivery System

The efficiency of drug delivery can be improved by combining the ability of immune cells to evade immune clearance with the homologous targeting of tumor cells. The two cell membranes can also synergistically activate the body’s immune activation to achieve tumor activation. Hao et al. developed a chemoimmunotherapy delivery vehicle based on C6 cell membranes and DC membranes to fabricate hybrid membrane-coated DTX nanosuspensions (DNS-[C6&DC]m) [140]. Membrane coating allows for immune escape and targeting of cancer cells using the homotypic targeting mechanism of C6 cell membranes. Meanwhile, after the hybridization of DC cell membranes and tumor cell membranes, dendritic cell membranes carry intact cancer cell membrane antigens, which can activate the immune response in the tumor microenvironment (Figure 9E,F). The ability of different immune cells such as immune evasion, capturing CTCs, and influencing TAM phenotypes provide more development directions for immune cell-cancer cell biomimetic nano-delivery systems. 

#### 3.6.4. Cancer Cell-Cancer Cell Biomimetic Nano-Delivery System

Different types of cancer cell membranes can prove useful for tumor therapy by integrating various properties through hybridization [142,143]. The blood–brain barrier presents a significant obstacle in the treatment of gliomas, necessitating delivery systems that can penetrate the barrier and precisely target gliomas. Chi et al. proposed a hybrid cell membrane (HM) camouflage strategy consisting of brain metastatic breast cancer cell membranes MCF-7 cell membrane (MM) and U87-MG cell membrane (UM). The nano-delivery system has BBB cross-over capability and homologous tumor-targeting capability inherited from two cell membranes of origin [141]. In vitro and in vivo investigations have been carried out on the ability of HMGINPs to cross the blood–brain barrier (Figure 9G). Trans-well experiments demonstrate that HMGINPs can cross the blood–brain barrier well simulation models. In vivo, fluorescence imaging results showed that HMGINPs acquired better blood–brain barrier penetration and homologous tumor-targeting ability compared with UMGINPs.

In summary, combinations between different cell membranes provide more diverse and creative directions for cell membrane-biomimetic nano-delivery systems for tumor therapy. A flexible combination of different cell membranes depending on the problem encountered in tumor therapy is a more promising technique.

## 4. Discussion

Inspired by living cell drug delivery, researchers have developed a CM-based biomimetic nano-delivery system to improve the oncology therapeutic effects of nanomedicines. These systems show excellent potential for cancer treatment applications including chemotherapy, immunotherapy, phototherapy, and in vivo imaging [144,145]. Different cells perform different tasks in the life activities of the organism and have many unique capabilities. Various CM coatings confer many interesting capabilities to nanomedicine delivery systems by retaining biomolecules on the membrane surface [146]. Cells applied in biomimetic nano-delivery systems developed so far include red blood cells, macrophages, dendritic cells, neutrophils, platelets, mesenchymal stem cells, cancer cells, and hybridized cell membranes. Relevant studies in recent years have shown that CMBNDS can address some of the problems facing oncology drug delivery (Table 2): (1) Utilizing the natural properties of biofilms to camouflage nanoparticles and improve the biocompatibility of nanoparticles; (2) Utilizing some cells that can remain in the blood for a long period, such as red blood cell membranes, platelet membranes, and leukocyte membranes, to avoid removal by physiological barriers and to prolong circulation time; (3) By surface coating some cell membranes with tumor-specific targeting, such as tumor cell membranes and platelet membranes, tumor-specific targeting of nanoparticles can be improved. (4) Cell membrane modification to enhance anti-tumor immunostimulation, such as tumor cell membranes, DC cell membranes, and their secretory membrane structures; (5) Engineered cells or cell membrane-modified nanoparticles to improve tumor-targeting ability.

Although CMBNDS has shown promising therapeutic prospects in cancer diagnosis and treatment, there are still some remaining problems in the development and clinical application of CMBNDS. Here we summarize the problems encountered in the development and clinical application of CMBNDS. First, source material is difficult to obtain. Although it is possible to obtain cells autologously, most of them still need to be matched to a suitable donor, and the cells need to be characterized, survived, tested for exogenous pathogens, and evaluated for their properties. In addition, according to GMP regulations, the process of collecting cell membranes should conform to the collection and isolation standardized operation management procedures and ensure that the phenotype of the cells remains unchanged during the passaging process. Second, the production technology of CMBNDS is relatively immature. There is a wide variety of preparation methods currently applied, and different methods operate under different conditions. There are no standardized principles for the selection of these approaches and procedures to achieve higher extraction efficiency. Besides, the large-scale preparation process route is cumbersome, the standard is not uniform, and the batch-to-batch reproducibility in mass production is poor. The third is the difficulty in characterizing CMBNDS. Current characterization methods only verify the success of the coating by particle size inspection and morphological appearance. Western blot can only demonstrate that the functional proteins of the NP surface are inherited, but whether the cell membrane structure is disrupted or not has not been studied in detail. Meanwhile, the mechanism of action for the presence of complex functional proteins and structural units on the surface of CMs remains unelucidated. Increasing the amount of desired functional proteins in the membrane may improve the therapeutic efficacy of CMBNDS. Fourth, the in vivo mechanism of cell membrane-based biomimetic nanoparticles, especially heterogeneous cell membranes, is unclear. It is difficult to clearly understand the in vivo processes in clinical trials. Finally, cell membranes are biological materials that require sterile storage conditions during preservation. In addition, membrane proteins should be kept stable to avoid unpredictable immune reactions. In summary, the safety and efficacy of CMBNDS require further studies to provide evidence and are still not ready for clinical application. The preparative methods for the scaled-up production of CMBNDS also need to continue to be refined to ensure that they are standardized and reproducible.

## 5. Conclusions

In conclusion, researchers have developed a variety of nano-delivery systems to solve the problems faced by traditional therapies, such as blood clearance, lack of drug targeting, poor tumor bioavailability, drug resistance, and side effects. However, nanotechnology in tumor therapy continues to be constrained by the non-specific binding of proteins in the blood, phagocytic clearance of MPS, and other biological barriers facing tumor therapy. The development of a cell membrane bionic nano-delivery system provides the nano-delivery system with unique biological properties of different cells, such as long circulation, good biocompatibility, escaping from immune cell clearance, and tumor-targeting in vivo. It can effectively deliver drugs to tumor tissues and provide a more effective development idea for tumor therapy. While there are still many issues to be resolved from the laboratory to the clinic, there are obvious natural advantages and application potential of CMBNDS. Continually combining technologies from other disciplines, such as biology and chemistry, can optimize the unique functions of cell membranes. The cell membrane biomimetic nanomedicine delivery systems will encounter greater development opportunities and bring a better future for tumor therapy.

## Figures and Tables

**Table 1:** Summary of Cell Membrane Extraction Methods.

Methods	Application	Advantages	Disadvantages	Refs.
Hypotonic lysis	Akaryote	Simple	Low efficiency	[46,47]
Ultrasonic	Karyocyte	Efficient	It affects membrane protein activity, hard to use on a factory scale	[48]
Repeated freeze–thaw	Akaryote	Simple	It affects membrane protein activity	[49,50]
Chemical lysis	Karyocyte	Efficient	The destruction of cell membranes is greater	[51,52]
Homogenizer	Karyocyte	Efficient, Suitable for largescale industry	Large energy consumption requires an enormous maintenance workload	[53,54]

**Table 2:** Function of cell membrane biomimetic nano-delivery system.

Type	Source Cell	Preparation Strategy	Function	Refs.
RBCNDS	Red blood cell	Coextrusion Sonication Electroporation	Long circulation Good biocompatibility	[71,76,77]
PNDS	Platelet	Coextrusion Sonication Electroporation	CTC-targeting; Low immunogenicity	[84,85,86,87]
ICNDS	Macrophage	Coextrusion Sonication	Tumor-targeting; Reducing opsonization; Transendothelial migration	[95,97,147]
	Neutrophil			[99,100]
	Dendritic cell			[106,148]
	T-lymphocyte			[111,113]
	Natural killer cell			[116,117]
MSCNDS	Mesenchymal stem cell	Coextrusion Sonication	Tumor-homing Low immunogenicity	[120,124,125]
CCNDS	Cancer cell	Coextrusion Sonication	Homologous targeting; Immune escape	[127,133,135]
HCNDS	RBC–platelet	Coextrusion Sonication	Long circulation Avoid immune clearance Target the inflammatory area	[138,149]
	RBC−cancer cell		Homologous recognition Homing characteristics Long circulation	[139]
	Leukocyte–cancer cell		Immune escaping Tumor-targeting	[140,150]
	Cancer-cancer		Transendothelial migration Tumor-targeting	[141]

## Data Availability

Data sharing is not applicable.
